# Application of Optical Biosensors in Small-Molecule Screening Activities

**DOI:** 10.3390/s120404311

**Published:** 2012-03-28

**Authors:** Stefan Geschwindner, Johan F. Carlsson, Wolfgang Knecht

**Affiliations:** 1 Discovery Sciences, AstraZeneca R&D Mölndal, 43183 Mölndal, Sweden; E-Mail: Johan.F.Carlsson@astrazeneca.com; 2 CVGI iMed, Bioscience, AstraZeneca R&D Mölndal, 43183 Mölndal, Sweden

**Keywords:** screening, fragment, ligand, biosensor, surface plasmon resonance, optical waveguide grating, drug discovery

## Abstract

The last two decades have seen remarkable progress and improvements in optical biosensor systems such that those are currently seen as an important and value-adding component of modern drug screening activities. In particular the introduction of microplate-based biosensor systems holds the promise to match the required throughput without compromising on data quality thus representing a sought-after complement to traditional fluidic systems. This article aims to highlight the application of the two most prominent optical biosensor technologies, namely surface plasmon resonance (SPR) and optical waveguide grating (OWG), in small-molecule screening and will present, review and discuss the advantages and disadvantages of different assay formats on these platforms. A particular focus will be on the specific advantages of the inhibition in solution assay (ISA) format in contrast to traditional direct binding assays (DBA). Furthermore we will discuss different application areas for both fluidic as well as plate-based biosensor systems by considering the individual strength of the platforms.

## 1. Introduction

Label-free optical detection systems for drug discovery and in particular for small-molecule drug screening have gained popularity during the past decade within industry and academia [1]. This is on one hand a direct consequence of the continuous development of novel and enhanced label-free detection systems showing improved assay robustness and better detection limits combined with increased throughput and multiplexing that matches the proliferating needs. On the other hand this is also a reflection of the increased demand of modern drug discovery to provide detailed biophysical ligand binding data like kinetic and thermodynamic information in addition to the more typical binding and/or affinity data [2–4]. Altogether this has positioned surface plasmon resonance (SPR) technology as a prominent approach to deliver orthogonal ligand binding information into the selection process e.g., during hit evaluation after a high-throughput (HTS) screen [5]. The adoption of SPR technology was further accelerated by increasing demand in efficient fragment-based drug discovery (FBDD) approaches [6,7]; this has made SPR technology a validated tool to detect small-molecule interactions.

The recent success of SPR technology has increased the interest and demand within big pharma industry for complementary platforms and technologies that could handle samples in a parallel high(er)-throughput fashion. Besides other alternative optical detection systems, the use of planar waveguide systems using microplates is seen as an interesting complement to SPR systems, as it enables small-molecule screening with increased throughput without compromising on data quality. Those optical waveguide grating (OWG) systems are either making use of resonant waveguide grating (Corning EPIC system) [8] or nanostructured optical grating (also known as photonic crystals as employed in the SRU BIND system) [9]. They have in common that they, in analogy to SPR-based approaches, are typically used to detect the binding of compounds to immobilised target proteins that are attached to a modified biosensor surface. The ability to attach even entire cells onto the biosensor surface and to follow the cellular responses upon challenge with a compound in real-time [10], opens up for novel and interesting screening approaches. However, this article will solely focus on biochemical assays and the challenges that are associated with those.

One of the main challenges in either using SPR- or OWG-based approaches relate to the used target protein. As already mentioned, a common approach is the tethering of the target protein onto the biosensor surface using a range of coupling chemistries and immobilisation strategies [11]. A key success factor for establishing a system that can be used for screening of larger compound libraries (*i.e.*, screening hundreds of compounds/day) is linked to the successful manipulation of the inherent instability of most target protein constructs, *i.e.*, the decrease in ligand-binding competence over time. This is particular important for microfluidic systems like the typical SPR-based Biacore instrument, as the screened compounds are frequently tested against the same sensor surface which needs to remain relatively constant in ligand binding activity during the entire series of experiments. There have been some intelligent approaches developed that either help to minimize or to compensate for the loss of ligand-binding activity during the course of a SPR screening campaign [7]. However, important drug targets like GPCRs, that frequently show a high intrinsic instability once extracted from the membrane, are frequently resilient to such approaches. Only dedicated efforts aiming to generate stabilised versions of some selected and well-behaving GPCRs (designated as stabilized receptors or STARs) enabled recently the successful screening against two important GPCR targets using SPR methodology [12,13]. This highlights the importance of having access to stable protein reagents. As OWG-based approaches are typically performed using plate-based systems, this becomes much less of an issue as the modified sensor surface is only used in a single binding experiment and typically gets disposed after its usage. This comes at the cost of an amplified target protein consumption that can be in the range of several orders of magnitude higher as compared to a fluidic SPR-based system and might exclude OWG-approaches for screening campaigns that have limited access to larger amounts of target protein.

A subsequent challenge relates to the reliability of detecting genuine binders during a screening campaign. This is very much dictated by the attainable sensitivity of the system as well as the ability to discriminate specific from unspecific binding events and is reflected in the rate of false negative and false positive hits respectively. The physical behaviour of compounds or fragments in aqueous solutions is quite frequently leading to situations where one can observe apparently specific binding events at lower compound concentration with a more dominant unspecific binding component at elevated compound concentrations. Usually one defines in advance a maximum binding signal that is expected for a 1:1 binding and uses that information to exclude compounds with unrealistically high stoichiometry [7]. Another common approach is to screen against a modified target protein, either by a site directed mutation or chemically modified, in the targeted binding site [5]. Thus performing concentration-response experiments will usually help to understand the mode of activity of such compounds better and help to remove aggregation-based compounds from the initial list of hits [14]. In contrast to that it is much more challenging to deal with false negative hits as this is very much determined by the sensitivity of the system. Here one needs to distinguish between the mass sensitivity that represents the characteristics of the sensor structure itself and is thus technology- and instrument specific, and the assay sensitivity that is additionally influenced by other factors such as the molecular weight of the interacting partners, their affinity as well as total concentration and density in the buffer and on the biosensor respectively. Simulations displaying the minimum required ratio between the total ligand concentration and the affinity as a function of the molecular weight of the ligand as well as the protein show, that assay sensitivity is lower with increasing molecular weight of the protein and decreasing molecular weight of the ligand [15]. This creates particular issues for studying low-affinity small-molecule fragment binding to larger protein systems, in particular if those are not displaying good ligand binding competence after the immobilisation to the biosensor. Thus different assay formats have been developed that can specifically address the issue of low assay sensitivity in particular for small-molecule work.

The direct binding assay (DBA) is widely used in kinetic analysis as well as screening studies [16,17]. It has the essential feature that compounds (the analyte) interact directly with the binding site of immobilised target proteins on the cost of low assay sensitivity as described earlier. By introducing a second analyte molecule that binds specifically to the same binding site and exhibits increased assay sensitivity due to its enhanced molecular weight; it will compete for the same binding site with the first analyte and thus serve as a well-observable reporter molecule for the binding of the first analyte [18,19]. As both analytes compete for the same binding site at the sensor surface this assay is also called surface competition assay (SCA). However, no binding reaction is taking place in solution. This is contrasted by the inhibition in solution assay (ISA) with the fundamental difference that binding of the analyte to the target protein is occuring free in solution [20,21]. The second analyte or an analogue thereof (often referred to as target definition compound or TDC) is tethered onto the biosensor surface and serves as a tool to determine the change in the free target protein concentration in the presence of the first analyte. This assay format was used particular in the early days of SPR more frequently as the mass sensitivity of those first instruments was not suitable to reliably quantitate small molecule binding to macromolecular targets. The enhancements in mass sensitivity seen particular during the last decade in the field of SPR has allowed studying those interactions directly. However, with the elevated FBDD efforts and thus fragment screening demand this assay format has clearly an enabling potential and has been already applied successfully in this context [22].

**Figure 1.**
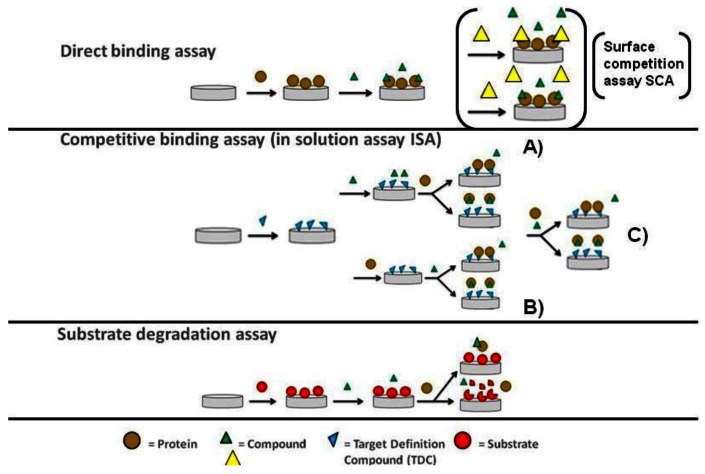
Assay formats. In the direct binding assay the target is immobilized directly on the biosensor. In brackets, a variant of the DBA, the so called surface competition assay (SCA) is shown. Both compounds compete for the same binding site at the sensor surface. In the inhibition in solution assay (ISA), binding of the analyte or compound to the target protein is occurring free in solution. In the ISA the order of addition of protein and competing small molecule can vary depending on the assay setup: (**A**) first addition of compound (**B**) first addition of protein (**C**) compound and protein has been pre-incubated. In the substrate degradation assay a mass change on the sensor surface is for example caused by an added protease digesting the protein immobilized on the sensor surface.

As previously stressed, OWG-based approaches are typically performed using plate-based systems and the sensor surface in each well is only used in a single binding experiment. This opens up for the development of more complex, more functional biochemical assays that are coupled to a mass change on the sensor surface on OWG platforms. A typical example for such an assay would be the proteolytic degradation of a protein substrate immobilised on the sensor surface by an added protease. A simple schematic outline of the three most common assay types that can be performed on optical biosensors in small molecule screening is shown in Figure 1.

In order to assess the value of the ISA format for small-molecule screening using optical detection systems, with the focus on novel high-throughput instruments as displayed by plate-based OWG systems, we conducted a comparative study using human trypsin as a model system on the SRU BIND and Corning Epic platform. The aim was to be able to directly compare the outcome of a defined screening set for a DBA, an ISA, a proteolytic assay performed on an OWG platform and a classical trypsin enzymatic assay using a small peptide as substrate. To our best knowledge, dedicated reports of the comparison of different assay formats on optical biosensors for small-molecule screening activities have not been published, yet.

The results from this practical example will guide us in discussing the usage and requirements of the ISA format in contrast to other assay formats as well as discussing some brief examples of its successful application in fragment screening.

## 2. Experimental Section

### 2.1. Assays

#### 2.1.1. Chromogenic Assay

To a compound plate containing 0.25 μL per well of compound (10 mM) in DMSO a volume of 40 μL trypsin I (Polymun Scientific) diluted in assay buffer (0.1 M Tris-HCl, 1 mM CaCl_2_, 0.1% (v/v) Tween-20, pH 8) was added. The plate was shaken and left to stand for 10 min. A volume of 10 μL Bz-Val-Gly-Arg-pNA (Bachem) substrate in assay buffer was then added to the compound plate to give a final substrate concentration at the K_m_ value of Bz-Val-Gly-Arg-pNA for trypsin I (360 μM). The increase in absorbance was then measured at 405 nm for 20 min at RT. Slopes were used to determine the inhibitory effects of the compounds. The chromogenic assay had a Z′ of 0.82.

#### 2.1.2. Biosensor Assays

Three different assay formats were run on biosensors integrated into microplates using the SRU Bind (SRU Biosystems) or the Epic platform (Corning): a DBA, an ISA and a substrate degradation assay with bovine serum albumin (BSA, Sigma). In addition a DBA with active site blocked trypsin I was also run to detect unspecific binders and used in combination with the DBA to trypsin I alone. The binding values to active site blocked trypsin were subtracted from the binding values to active trypsin I. Active site blocked trypsin I was obtained by treating trypsin I with the irreversibly binding substrate analog FPR-CMK (Sigma). In the ISA, biotinylated-FPR-CMK (Haematologic Technologies) was immobilized to the biosensor plate as bait. The characteristics of the four resulting biosensor assays are summarized in Table 1.

For immobilization of trypsin I or active site blocked trypsin I, 5041 plates from Corning were used. They contain pre-activated chemistry based on polymeric maleic anhydride groups. Proteins are bound by covalent (amide) bond formation with NH_2_ groups. BSA for the degradation assay was adsorbed to unmodified titaniumoxide coated biosensors in the microplates. Biotinylated-FPR-CMK was bound to pre-made streptavidin coated plates (SA-1) from SRU Biosystems.

### 2.2. Screening

A test set of 323 compounds was selected from AstraZeneca's screening database on the basis of previous determined IC_50_ values available for human trypsin I. Further criteria for selection were M_w_ < 500 Da, LogD < 5, solubility > 10 μM and finally a visual inspection of the compound structures by a medicinal chemist. The compounds were then divided into three subsets (Table 2), depending on their previously found activity.

The set was run three times independently in each assay at a single concentration of 50 μM. A difference of at least 6 × SD from the background control in at least two of the three assay occasions was used as criterion to define a hit (=Active A). 100% effect was defined by an AstraZeneca developed trypsin inhibitor and used to define unspecific binding of compounds in the direct binding assays. All binding signal in the direct binding assays were corrected for the M_W_ of the compound and the immobilization of trypsin in the respective well.

## 3. Results and Discussion

**Figure 2.**
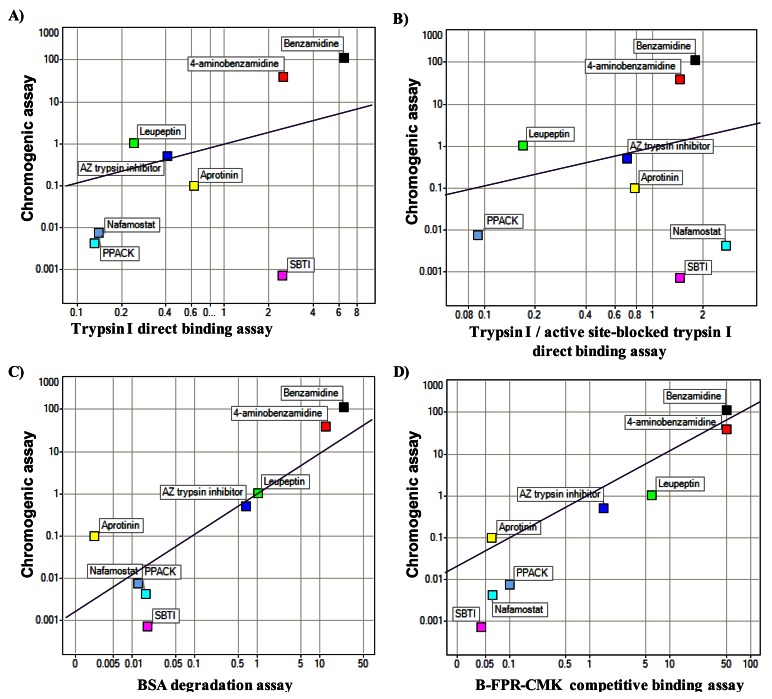
Comparison of the chromogenic assay with the optical biosensor based assay formats for a selection of small molecule and protein inhibitors. IC_50_ values for the chromogenic assay are in μM, for the DBA in (**A**) and (**B**) K_d_ values are given in μM, for the substrate degradation assay (**C**) and the ISA (**D**), IC_50_ values are given in μM. The solid lines show a 1:1 correlation.

Proteases are a common drug target. Human trypsin I was chosen as model protein to test and compare optical biosensor based assay formats for protease inhibitor discovery: DBA, ISA and substrate degradation assay (see material and methods for details as well as Figure 1). The results from these were compared to those generated with a chromogenic enzymatic assay based on cleavage of a small peptide. The different assay formats were first compared to the chromogenic assay by the determination of the K_d_ or IC_50_ values for a selection of small molecules and protein inhibitors. The results are shown in Figure 2.

It seems that the protein protease inhibitor SBTI and the small molecule compound nafamostat are outliers in the DBA assay formats by ranking, whereas the small molecules in general seem to correlate well to the chromogenic assay (Figure 2). From the determination of K_d_ and IC_50_ values it was seen that the different assay formats differed in sensitivity, e.g., the ISA being less sensitive than the substrate degradation assay. However, this is in line with the higher amount of trypsin I used in this assay format (Table 1).

We then compared the different assay formats with a larger set of compounds at one concentration as one would typically carry out in a primary screening effort to find new hits. However we composed a test set to incorporate a sufficiently high number of active compounds (desired hits) as well as non-actives and frequently hitting compounds based on previous screening experience with trypsin in our company. The results from this comparison are shown in Table 3. The substrate degradation and the ISA delivered results showing a good correlation to the chromogenic enzymatic assay. The DBA however showed a considerable different outcome for the test set. The DBA delivered many compounds of the Non-active and Frequent hitters subset as hits (Table 3). The number of these false positives could be decreased by using active site blocked trypsin as control. However as a normal screening set in contrast to our selected test set is composed of mainly Non-actives; a 24% hit rate is still way too high. The DBA assay would therefore in reality deliver an overhelming number of false positives when compared to the ISA or degradation assay. This rate could be even higher, if no caution is taken to sort out unspecific binding compounds in the DBA setup. As it can be seen in Table 3, a main reason for exclusion as real hit (N) is detection of unspecific binding. Surprisingly also a substantial number of compounds expected to be found as hits (as originating from the Actives subset) in the DBA showed unspecific binding, raising questions about their mechanism of action in the other assay setups.

These reflections promote the concept that if microplate-based biosensors should be used in a primary screening setting, ISA or a substrate degradation assay should be first considered. Additionally, in these assays, the target protein is in solution in contrast to the DBA, where the protein is randomly immobilised to a surface at very high local density. This is in line to minimize the number of false positive hits to be obtained in total. However, it must kept in mind that in the ISA and degradation assay format also additional methods are required to then filter out compound hits with an undesired mode of action (unwanted positives), e.g., unspecific binding or aggregating compounds. But again, considering total numbers, testing a small number of active hits delivered by an ISA or degradation assay with an orthogonal assay format for unwanted mode of action is more efficient than sorting out false positives from an overhelming number of hits as the DBA would deliver.

**Figure 3.**
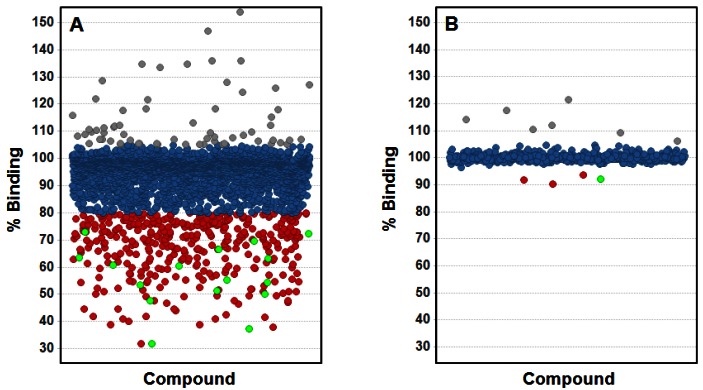
OWG ISA screening results on two targets with different ligandability. Activity is reported as % Binding, *i.e.*, the binding of the target protein to the TDC-modified biosensor in presence of compound in relation to the controls containing only target protein. Non-actives are depicted in blue, Actives (as defined by a cut-off value) are depicted in red and Actives with positive NMR-binding results are depicted in green. Compounds that interfere with the readout due to aggregation or solubility issues are depicted in grey. (**A**) Shown is the fragment screening result for the target possessing high ligandability; (**B**) Shown is the fragment screening result for the target possessing low ligandability.

The results obtained with trypsin as a model system indicate a good quality performance of the ISA format using plate-based OWG systems for small-molecule screening, in particular considering the ability to identify true binders as well as the low rate for false positive binders. In order to further evaluate the usefulness of this assay format for fragment screening utilizing OWG platforms, we conducted a fragment screening campaign on an internal drug target with high ligandability (*i.e.*, high probability of finding small molecule inhibitors, see [23]) as well as a drug target with low ligandability, in this case a protein-protein interaction (PPI). In order to validate the output from both screens we applied biomolecular NMR techniques, which are seen as a golden standard due to their superior detection sensitivity [1]. A set consisting of about 3,000 fragments was screened against the target displaying high ligandability using the ISA format on the SRU BIND system utilizing a specifically designed TDC (see Figure 3(A)). The screen was performed at 100 μM compound concentration and returned 395 fragment hits (=13.2% hit rate) thus demonstrating a hit rate in line for a drug target with high ligandability. From the initial hit set, 16 fragments presenting interesting chemotypes have been selected for further analysis employing NMR spectroscopy, and all 16 fragment hits could be verified as competitive molecules binding to the drug target indicating a very high verification rate.

The PPI target was screened in a parallel fashion by means of NMR as well as applying the OWG ISA format and was challenged with a set of about 800 fragments at 60 μM and 100 μM concentration respectively. Both the NMR screen as well as the OWG ISA returned four fragment hits (=0.5% hit rate) thus delivering a hit rate in line with expectations (see Figure 3(B)). Interestingly, one fragment could be identified in both approaches that also displayed the highest affinity. The other three NMR hits have been outside the range of detection for the ISA due to the incompatibility of their mM-affinity with the selected screening concentration of 100 μM. The other three fragments hits from the ISA are likely binding to the TDC and are thus displaying false positive hits, albeit at a very low rate.

The successful application of the ISA method in real screening projects indicate a great potential of this approach for small-molecule screening using biosensors in terms of matching the needs for high-throughput screening with good assay sensitivity and robustness, but it has obviously some shortcomings that need to be considered as well. A key element of the ISA is the availability of a suitable TDC–typically such molecules are not readily available and need to be specifically designed to match available immobilisation chemistries as well as to exhibit still good affinity (typically sub-μM) to the target protein once immobilised. Some knowledge about the 3-dimensional structure of the target protein in conjunction with available substrates, ligands or compounds can be of tremendous help for the rational design of such tool compounds. For instance, the choice for the optimal position and length of the chemical linker that is used for tethering onto the biosensor can be greatly facilitated by structural information. The opportunity for docking of such molecules allows making informed judgements on the potential effects of the linker for the binding mode of the tool compound. Another interesting aspect is the possibility to use the TDC for the screening of other proteins within the same protein family, thus leading to the build-up of a target-family specific toolbox that is readily available for the screening of novel targets within the same protein family.

As opposed to the DBA, the ISA will only give information about compounds that are in direct kinetic competition with the TDC or alter the affinity to the TDC by binding to an orthosteric site. Thus, the assay format will not help to identify compounds that bind to other potential binding sites, but will instead deliver direct information about the binding site specificity and location. As such, the assay can serve as a standard secondary screening setup in situations where different modes of binding need to be considered and the primary screening results needs to be scrutinised for competitive compounds. Comparing with the information obtained from fluidic-based technologies like SPR it is also worth to make the observation, that the information content tends to be larger due to the availability of kinetic binding information. This kind of information can obviously not be accessed from OWG platforms due to its plate-based nature making use of equilibrium binding data. Interestingly, the ISA format if used in the mode of adding a compound to an already equilibrated solution containing the target protein, can theoretically provide access to kinetic data as this information is actually contained in the decay profile that is typically seen when displacing the protein from the biosensor. A related approach has been described using fluorescently-labelled probes indicating a good feasibility to extract that information from time-resolved binding data from ISA experiments [24].

The utilization of biological reagents and consumables during larger screening campaigns is in fact a key factor influencing cost efficiency and can actually set limits for the screening of larger compounds libraries. The current experience with performing biochemical assays on OWG platforms in a DBA format is such that approximately 0.5–1 mg of target protein is required for immobilisation using a 384-well plate. As those plates are typically not regenerated after screening it is apparent that it will be impractical to screen a full HTS deck which typically comprises between 1–2 million compounds. Using the ISA format instead furnishes the opportunity to control the amount of target protein used for screening as this is very much determined by the affinity of the TDC. Furthermore the plates can be easily regenerated during the screening campaign, as most TDCs are compatible with applying relatively harsh conditions during regeneration, and can thus be recycled in the screening process.

## 4. Conclusions/Outlook

The recent introduction of microplate-based OWG platforms triggered us to assess the impact of an assay format that has been intensively used particular during the period after the launch of the first commercial SPR platform in 1990, on the success for small-molecule screening—the inhibition in solution assay (ISA). In summary, the above outlined experiences with ISA on OWG platforms point towards distinct advantages of this assay format for fragment screening as well as orthogonal screening for hit validation, where higher throughput screening capacity is required.

In addition, we believe, the potential to use more functional biochemical assays on microplate-based OWG platforms, as exemplified here by the substrate degradation assay, has not yet been appreciated and exploited fully. Currently we are investigating further functional assays, e.g., buffer clot lysis assays to study coagulation factors, on OWG platforms, thus trying to reduce the gap between *in vitro* and *in vivo* assays leading to the identification of biologically relevant molecules.

## Figures and Tables

**Table 1.** A summary of the assay conditions of the assay formats on the microplate biosensors specifying microplates used, immobilization conditions and Z′ values of the resulting assays.

**Assay format**	**DBA**	**DBA**	**Degradation Assay**	**ISA**
Immobilised on biosensor	Trypsin I	Trypsin I & active site blocked trypsin I	BSA	Biotinylated-FPR-CMK
Biosensor	5041	5041	TiO_2_	SA-1
Platform	Epic	Epic	SRU BIND	SRU BIND
Immobilization buffer	20 mM Na-acetate pH 5	20 mM Na-acetate pH 5	20 mM Hepes pH 8	20 mM Hepes pH 7
Immobilization concentration	100 μg/mL	100 μg/mL	50 μg/mL	3 μM
Time of immobilization	Overnight	Overnight	Overnight	30 min
Trypsin I addition	-	-	1 μg/mL	10 μg/mL
Z′	0.66	0.67	0.38	0.66

**Table 2.** Subsets of the test set. The selected compounds were divided into three different subsets depending on their previously found activity as outlined under description.

**Subset**	**Number of Compounds**	**Description**
Actives	171	Active compounds based on AstraZeneca data from previous trypsin assays. IC_50_ of less than 10 μM.
Non-actives	96	Compounds that showed no activity in the previous trypsin assays.
Frequent hitters	56	Compounds that have been tested in at least 40 assays and that have appeared as active in at least 60% of these.

**Table 3.** Summary of the screening results for all assays. The results for the test set subsets are reported in % of the total. For the DBA, compounds showing unspecific binding (either high values compared to the expected maximum binding signal or similar binding to both trypsin I & active site blocked trypsin I) were classified as not being hits (N) and their % of the total is given in brackets. Hits are reported as A.

**Assays**	**Subsets**					

	**Actives**		**Non-Actives**		**Frequent Hitters**	
	N	A	N	A	N	A
Chromogenic assay	15	85	100	0	98	2
DBA (Trypsin I)	56 (50)	44	55 (14)	45	72 (55)	28
DBA (Trypsin I–active site blocked Trypsin I)	58 (47)	42	75 (25)	24	89 (71)	11
ISA	27	73	99	1	93	7
Degradation assay	42	58	100	0	98	2

## Abbreviations

DBA: direct binding assay
FBDD: fragment-based drug discovery
HTS: high throughput screening
ISA: inhibition in solution assay
NMR: nuclear magnetic resonance
OWG: optical waveguide grating
SCA: surface competition assay
SPR: surface plasmon resonance
TDC: target definition compound
